# Polymeric Core-Shell Nanoparticles Prepared by Spontaneous Emulsification Solvent Evaporation and Functionalized by the Layer-by-Layer Method

**DOI:** 10.3390/nano10030496

**Published:** 2020-03-10

**Authors:** Marta Szczęch, Krzysztof Szczepanowicz

**Affiliations:** Jerzy Haber Institute of Catalysis and Surface Chemistry, Polish Academy of Sciences, Niezapominajek 8, PL-30239 Krakow, Poland; nclapczy@cyf-kr.edu.pl

**Keywords:** drug delivery, polymeric nanoparticles, core-shell, magnetic targeting, passive targeting

## Abstract

The aim of our study was to develop a novel method for the preparation of polymeric core-shell nanoparticles loaded with various actives for biomedical applications. Poly(caprolactone) (PCL), poly(lactic acid) (PLA) and poly(lactide-co-glycolide) (PLGA) nanoparticles were prepared using the spontaneous emulsification solvent evaporation (SESE) method. The model active substance, Coumarin-6, was encapsulated into formed polymeric nanoparticles, then they were modified/functionalized by multilayer shells’ formation. Three types of multilayered shells were formed: two types of polyelectrolyte shell composed of biocompatible and biodegradable polyelectrolytes poly-L-lysine hydrobromide (PLL), fluorescently-labeled poly-L-lysine (PLL-ROD), poly-L-glutamic acid sodium salt (PGA) and pegylated-PGA (PGA-g-PEG), and hybrid shell composed of PLL, PGA, and SPIONs (superparamagnetic iron oxide nanoparticles) were used. Multilayer shells were constructed by the saturation technique of the layer-by-layer (LbL) method. Properties of our polymeric core-shell nanoparticle were optimized for bioimaging, passive and magnetic targeting.

## 1. Introduction

Numerous new chemicals have been developed to treat various complicated diseases effectively, but at the same time, some of them produce serious side effects, proving that the benefit does not always outweigh the risk [1,2]. Moreover, more than 40% of pharmacologically active compounds exhibit poor solubility in water. However, they have been proven to be very effective in vitro but cannot reach the side of action deeming them nearly worthless in vivo. While great progress has been made in identifying drug targets along with designing and making better drug molecules, there is still room to improve the drug-delivery systems and targeting [1,3]. Currently, we have a broad set of pharmaceutical nanocarriers (liposomes, micelles, solid lipid nanoparticles, niosomes, dendrimers, polymeric nanoparticles, etc.) at our disposal, along with the means to load them with various drugs and adjust their surface properties to make them long-circulating, targeted, stimuli-sensitive, and even multifunctional [4]. Polymeric nanoparticles have become an important area of research in the field of drug delivery. The early nanoparticles were mainly formulated from poly(alkylcyanoacrylate), currently the most widely used polymers for nanoparticles have been: from natural proteins or polysaccharides e.g., chitosan, alginate; and synthetic polymers e.g., poly(lactic acid) (PLA), poly(glycolic acid) (PGA), and their copolymers, poly(lactide-co-glycolide) (PLGA) [1,5,6,7,8]. These polymers are known for both their biocompatibility and resorbability through natural pathways [7]. The initial therapeutic effect of drug-loaded nanoparticles was relatively poor due to rapid clearance of the particles by phagocytosis post-intravenous administration. In recent years, this problem has been solved by the proper surface modification of nanoparticles e.g., by grafting their surface with many hydrophilic and flexible polymers. Presently, polyethylene glycol (PEG) is the polymer most often used for nanomaterial functionalization. Alternative strategies replacing PEG with poly-amino acids, e.g., poly-L-glutamic acid (PGA) has been also implemented lately [9,10,11]. One of the powerful methods of surface modification is the sequential adsorption of charged nano-objects called the layer-by-layer (LbL) method [11,12,13,14]. Advantages of the LbL method is the ease of manipulation and the multifunctionality that comes from the possibility of modification of the multilayer shell by organic molecules, polymers, inorganic nanoparticles, carbon nanotubes, antibodies, [15] by the introduction of functional groups [16], lipids [17] or nanoparticles [18]. That multifunctionality can be utilized for the preparation of targeted drug delivery systems. Targeting can be broadly classified into three regimes; passive, active and physical one [19]. In the frame of physical pH-sensitive, temperature-sensitive, redox potential-sensitive, ultrasound-sensitive, magnetic-sensitive systems are included. Two of them: the passive targeting and physical one with magnetic-sensitive systems are widely studied. Passive drug targeting based on an accumulation of drug through leaky vasculature of a diseased area. It was found that under certain pathological states, such as tumors, infarcts, and inflammation, the permeability of vascular endothelial increases and they become leaky. In such regions with increased vascular permeability, nanoparticles can accumulate and exert their therapeutic effect. This phenomenon is also known as an ‘enhanced permeability and retention’ (EPR) effect [19,20,21]. The concept is based upon conjugation of a drug molecule or drug nanocarriers with magnetic particles and guiding and concentrating them towards the intended pathology site under the influence of an external magnetic field [22,23,24,25,26]. In magnetic-sensitive systems, iron oxide nanoparticles referred to as superparamagnetic iron oxide nanoparticles (SPIONs) with particle size 4–10 nm are used.

Nanoparticles (NPs) were initially developed as drug carriers, but they also received attention as carriers of diagnostic agents. Moreover, both modalities, therapeutic and diagnostic can be on one platform called theranostics. Theranostic nanoparticles may simultaneously monitor and treat disease. Among the available imaging modalities including optical imaging (OI), magnetic resonance imaging (MRI), computed tomography (CT), ultrasound (US), positron emission tomography (PET) or single-photon emission computed tomography (SPECT), each has its own unique advantages [27,28,29].

Not only size but also size distribution, shape and surface of nanoparticles dictate their interactions with biological systems. Therefore, all of them should be taken into account when designing nanoparticles for drug delivery and imaging [30,31].

**Figure 1:**
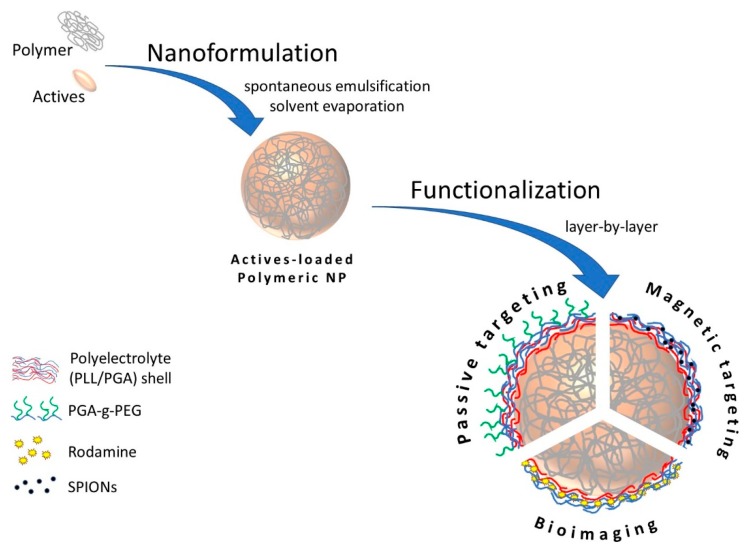
General scheme of proposed approach.

Based on the aforementioned data, the aim of the present study was to develop a novel method of preparation of loaded polymeric core-shell nanoparticles for biomedical application. Polymeric nanoparticles were prepared by the spontaneous emulsification solvent evaporation (SESE) method with the following polymers: poly(caprolactone) (PCL), poly(lactic acid) (PLA) and poly(lactide-co-glycolide) (PLGA). The model active substance was encapsulated in formed polymeric nanoparticles, then they were modified/functionalized by the LbL method for bioimaging, passive and magnetic targeting (Figure 1).

## 2. Materials and Methods

### 2.1. Chemicals

Polymers: polycaprolactone (PCL, average Mw ~14,000), poly(lactic acid) (PLA, average Mw ~60,000), and copolymer poly(lactide-co-glycolide) (PLGA, average Mw 4,000 to 15,000), polyelectrolytes: poly-L-lysine hydrobromide (PLL, average Mw 15,000 to 30,000), poly-L-glutamic acid sodium salt (PGA, average Mw 15,000 to 50,000), Coumarin-6, docusate sodium salt (AOT), sodium chloride (NaCl), lissamine rhodamine B sulfonyl chloride (ROD) were received from Sigma-Aldrich (Poznan, Poland). Superparamagnetic iron oxide nanoparticles (SPIONs) were acquired from PlasmaChem (Berlin, Germany. Chloroform was purchased from Avantor Performance Materials (Gliwice, Poland) while ultra-purified water was obtained using the Direct-Q 5UV purification system from Millipore (Warsaw, Poland). All chemicals were used without further purification. Chemical structures of the compounds are available in the Supporting Materials (Figure S1).

Pegylated polyanion PGA-g-PEG (g ~30% and PEG, Mw ~5000) was previously synthesized in our lab [32] while fluorescently-labeled poly-L-lysine hydrobromide (PLL-ROD) was synthesized via coupling of lissamine rhodamine B sulfonyl chloride according to the protocol described in [33].

### 2.2. Polymeric Nanoparticles’ Preparation

Polymeric nanoparticles were prepared by the SESE method [6,8] with some modifications. Briefly, certain amounts of polymer and surfactant AOT were dissolved in a solvent mixture comprising chloroform and ethanol, and then the mixture was gently added into an aqueous solution containing polycation (PLL) under stirring (500 rpm). After the formation of a stable nanoemulsion, the organic solvent was evaporated either by increasing the temperature/under vacuum or by continuous stirring to finally form polymeric nanoparticles. Blank nanoparticles were prepared following a similar procedure.

### 2.3. Drug Encapsulation and Efficiency of Encapsulation (EE)

Drug-loaded polymeric nanoparticles were prepared according to the procedure described above. Briefly, prior emulsification, selected drug at a various concentration ranging from 0.15 to 3 mg/mL was dissolved in the organic phase. To evaluate encapsulation efficiency, synthesized polymeric NPs were ultracentrifuged and the amount of free unencapsulated drug in supernatant solution was evaluated. Efficiency of encapsulation was calculated by the following formula:

(1) $$EE=\frac{\left|drug\right|total-\left|drug\right|supernatant}{\left|drug\right|total}\times 100\%$$

where |drug|total—the total weight of drug and |drug|supernatant—the weight of the free drug in the supernatant (not encapsulated).

### 2.4. Modification and Functionalization—Formation of Polymeric Core-Multilayer Polyelectrolyte Shell Nanoparticles

Prepared polymeric nanoparticles were further modified/functionalized by the multilayer shell formation. The shell was formed by the layer-by-layer method using the saturation approach. A fixed volume of polymeric NPs was added to the oppositely charged polyelectrolyte’s solution during vigorous mixing and the consecutive layer formation was followed by the zeta potential measurements. Then, the coating process was repeated with the use of the oppositely charged polyelectrolyte. The multilayer shell was constructed from the following charged nano-objects: PLL, PLL-ROD or SPIONs as the cationic one, and PGA as the anionic one. To create pegylated polymeric nanoparticles, PLL-terminated polymeric core-shell NPs were coated with the layer of PGA-g-PEG using the same procedure as described above. As a result, polymeric core-shell nanoparticles were prepared.

### 2.5. Nanoparticles’ Characterization

All synthesized polymeric nanoparticles were characterized by measurements of their size, size distribution, zeta potential, concentration, and morphology observation. Fluorescent emission spectra of the loaded polymeric NPs, as well as empty ones, were acquired to confirm drug encapsulation. For evaluation of the stability of synthesized polymeric nanoparticles, their size was monitored in time during storage in the preparation buffer.

The zeta potential, average size and size distribution of synthesized polymeric nanoparticles and their coatings with a various number of layers were carried out on a Zetasizer Nano ZS instrument (Malvern-Pananalytical, Malvern, UK). Additionally, the concentration and the size distribution of polymeric NPs were determined using Nanosight NS500 instrument (Malvern-Panalytical, Malvern, UK). The morphology of polymeric nanoparticles was analyzed by a cryo-scanning electron microscope (cryo-SEM) Jeol JSM-7600F Field Emission Scanning Electron Microscope, FESEM (Jeol Ltd., Tokyo, Japan) according to the protocol described previously [34]. Spectrofluorimetric measurements were acquired using a HORIBA Jobin Yvon Fluorolog-3 spectrofluorometer equipment (HORIBA Jobin Yvon, Longjumeau, France).

## 3. Results and Discussion

### 3.1. Polymeric Nanoparticles—Synthesis and Characterization

**Figure 2:**
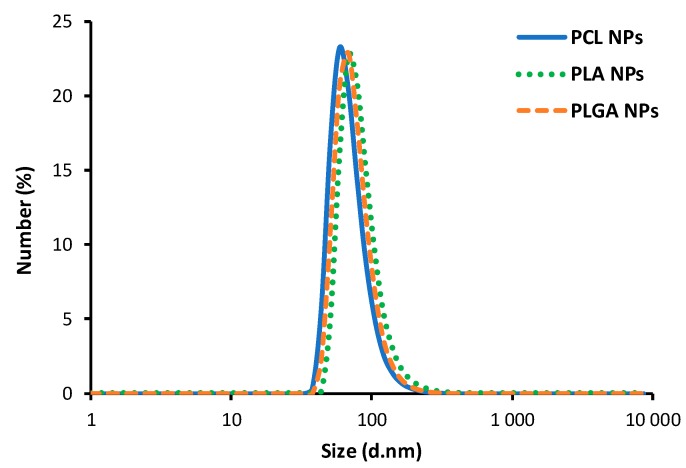
Size distribution of PCL, PLA and PLGA nanoparticles measured by dynamic light scattering (DLS).

**Figure 3:**
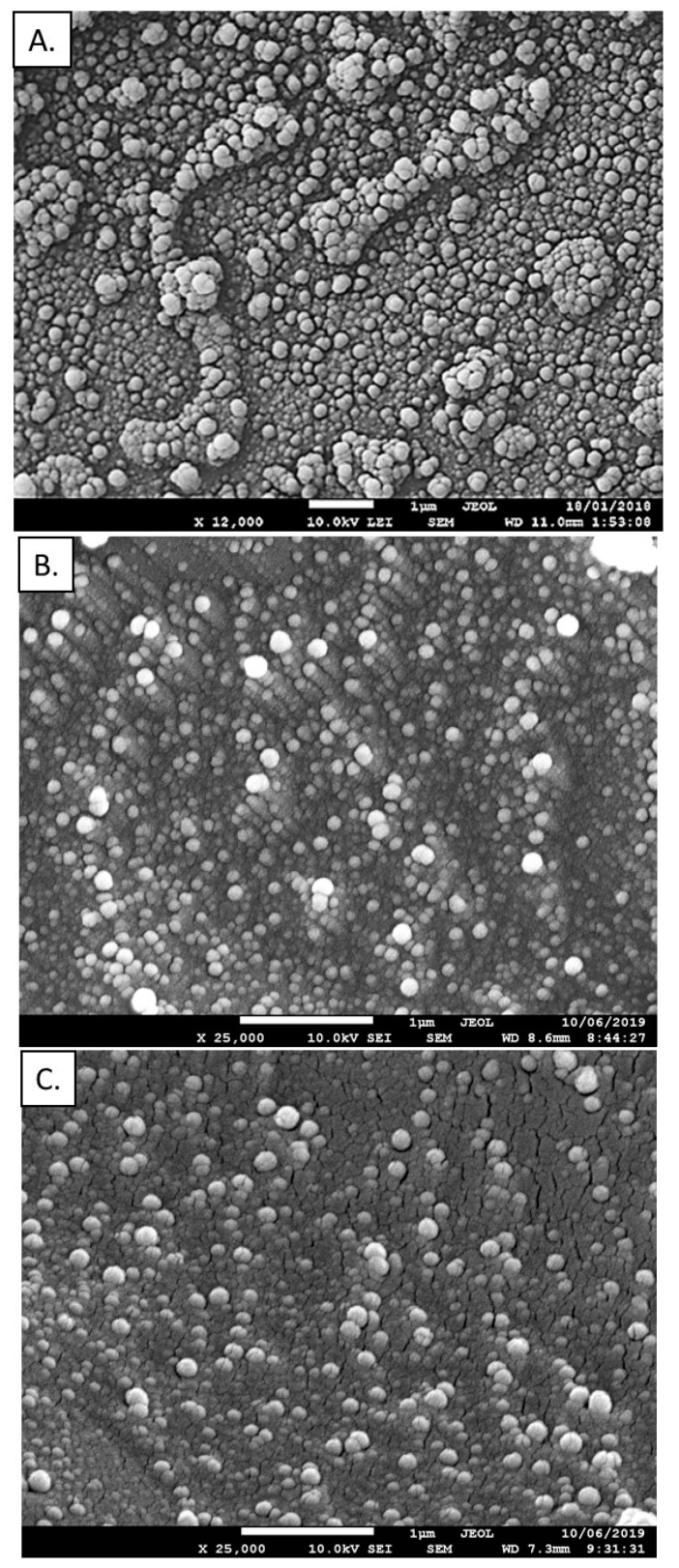
Cryo-scanning electron microscope (cryo-SEM) images of obtained PCL (**A**), PLA (**B**) and PLGA (**C**) nanoparticles.

In the emulsification/solvent evaporation technique selected polymer is dissolved in an organic solvent and this mixture is then dispersed into an aqueous solution to make oil in water nanoemulsion by using a surfactant agent. After formation of the stable nanoemulsion, the organic solvent is evaporated leading to formation of polymeric nanoparticles [8]. The high energy emulsification methods like high-speed homogenization or sonication are commonly used for nanoemulsion preparation, therefore, in our work, we decided to use an alternative approach, low-energy emulsification method [34], nanoemulsification assisted by Ouzo effect. From the list of biodegradable and bioacceptable polymers, the following were selected for our work: poly(caprolactone) (PCL), poly(lactic acid) (PLA) and poly(lactide-co-glycolide) (PLGA). The oil phase was prepared by dissolving the selected polymer in the easily evaporating solvent, chloroform, with the addition of anionic, oil-soluble surfactant AOT. The concentration of the surfactant favorable nanoemulsification was chosen from our previous work [35], and it was 33% w/v, while the concentration of the selected polymer in organic solvent was varied from 2.5 to 25 mg/mL. Such prepared oil phase was mixed with absolute ethanol (0.1 mL of the oil phase and 10 mL alcohol) and then gently added into an aqueous solution containing polycation (PLL) under stirring (500 rpm). Since the formed nanoemulsion is stabilized by the AOT/PLL interfacial complex, the amount of PLL was also optimized to minimize free, unadsorbed polyelectrolyte, which is obligatory for further modification by the layer-by-layer method (Supporting Materials (SM) Figure S2). The final step of the preparation was evaporation of an organic solvent either by increasing temperature/in vacuo or by continuous stirring. Both approaches result in similar sizes of polymeric nanoparticles, however, evaporation by the continuous stirring result in lower polydispersity index (PDI). Optimized parameters including drug and polymer concentrations are summarized in the Supporting Materials as Table S1. Characterization of polymeric nanoparticles formed under optimized conditions are summarized in Table 1, while the example of size distribution measured by dynamic light scattering (DLS) and cryo-SEM images for most favorable ones are presented on Figure 2 and Figure 3, respectively.

**Figure 4:**
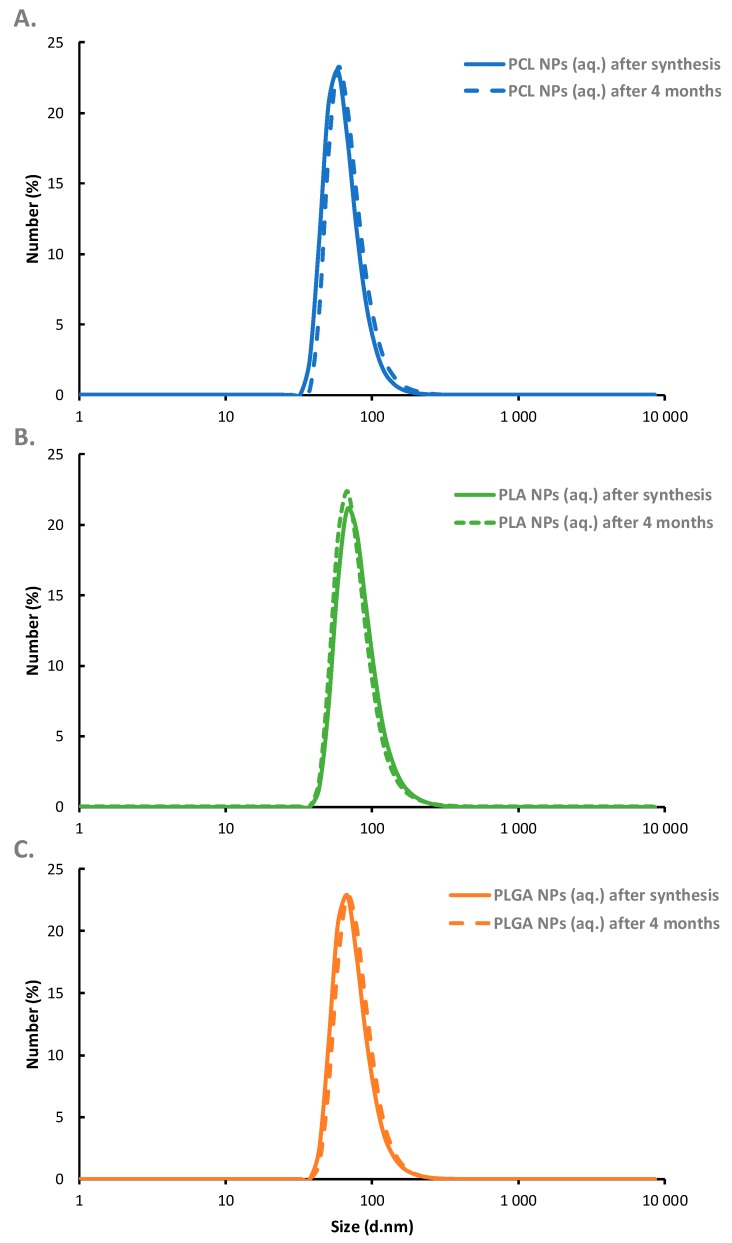
Long-term stability of synthesized PCL (**A**), PLA (**B**), and PLGA (**C**) nanoparticles evaluated by size-distribution measurements.

As can be seen in Figure 2, obtained sizes are independent on the type of polymer which indicates that the final size of synthesized polymeric nanoparticles is governed by the size of nanoemulsion droplets used as a template for nanoparticles’ synthesis. Optimization of the preparation parameters allow the formation of polymeric nanoparticles with average size ranged from 70 to 80 nm, with a polydispersity index (PDI) below 0.2, indicating that monodisperse polymeric NPs could be produced under optimized condition. It is also revealed that the average surface zeta potential of the formed polymeric nanoparticles was +72 mV (±4 mV) which provides the capability of further functionalization by the layer-by-layer method and is high enough to ensure electrostatic stability of the systems. Therefore, systematic measurements of the average size and zeta potential of synthesized polymeric NPs confirmed the long-term stability of the tested systems. It can be summarized that our nanoparticles were stable for at least four months (Figure 4). The concentration of synthesized polymeric NPs determined by NTA (nanoparticle tracking analysis technique) for all nanoparticle types was ~1 × 10^11^ nanoparticle/mL.

### 3.2. Active’s Encapsulation

Among numerous pharmaceuticals, some are especially difficult to handle because of their poor solubility in water-based biological fluids. Poor solubility results in poor bioavailability and difficulty in maintaining drug therapeutic concentrations in the blood [4,36]. Therefore, a hydrophobic fluorescent dye Coumarin-6 (C-6) was selected as a model active substance for our development. Polymeric nanoparticles containing Coumarin-6 were prepared according to the procedure described above. Prior to emulsification, Coumarin-6 was dissolved in the oil phase. To optimize the procedure, the concentration of the model dye was varied from 0.15 to 3 mg/mL, and the optimal amount was obtained when a signal from Coumarin-6 in supernatant solution after ultrafiltration was not observed. Efficiency of encapsulation for the optimized condition was calculated and summarized in Table 2. Characteristic emission spectra of Coumarin-6 loaded polymeric nanoparticles’ suspension (peak at ~500 nm, see SM Figure S3) can be observed and comparison of spectra of empty and loaded polymeric NPs provided the evidence of successful encapsulation of the model substance.

### 3.3. Functionalization of Polymeric Nanoparticles—Multilayer Shell Preparation

The layer-by-layer adsorption of charged nano-objects is considered as a convenient method to obtain core-multilayer polyelectrolyte shell colloidal particles and those types of particles have been the subject of intensive research since their invention in 1998 by Shukorukov [12]. The main advantage of the layer-by-layer method is simplicity of manipulation and the multifunctionality that comes from the possibility of modification of the polyelectrolyte shell by various functional species [15,16,17,18]. That multifunctionality can be utilized for the preparation of targeted drug-delivery systems. Therefore, synthesized polymeric nanoparticles were modified by the LbL method. Three types of multilayered shell were formed: two types of polyelectrolyte shell where biocompatible and biodegradable polyelectrolytes poly-L-lysine hydrobromide (PLL), fluorescently-labeled poly-L-lysine (PLL-ROD), poly-L-glutamic acid sodium salt (PGA), and pegylated-PGA with PGA-g-PEG were used; and a hybrid shell where PLL, PGA, and SPIONs were used. Multilayer shells were constructed by saturation technique of the LbL method. In the saturation technique, the rinsing step is omitted since it is possible to add just enough charged nano-objects to completely coat all of the particles present in the system so that there are little free unadsorbed nano-objects remaining in the aqueous phase [37]. This is the most important advantage of the saturation technique because the rinsing of nanoparticulate systems is problematic and associated with nano-objects’ loss.

In our research for each system, the saturation concentration was determined empirically by monitoring changes of the zeta potential of polymeric nanoparticles during mixing with oppositely charged nano-objects. The optimal amount was determined and corresponds to the point just before reaching the plateau of the dependence of zeta potential on the added amount of charged nano-objects and the value was usually close to the zeta potential of nano-objects in solution/suspension [38]. Since the results obtained for PCL, PLA, and PLGA were comparable for the following study, PCL nanoparticles were selected as a representative one.

#### 3.3.1. Polymeric Core-Shell Nanoparticles for Passive Targeting

**Figure 5:**
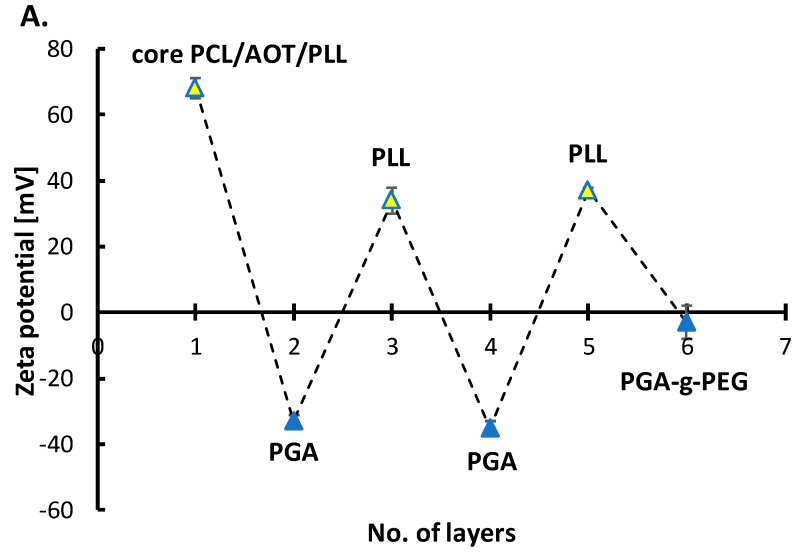

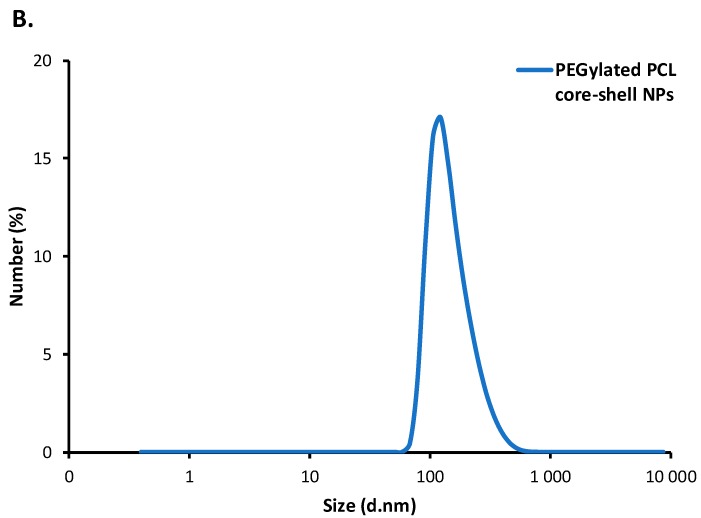
(**A**) Dependence of the zeta potential of polymeric core-shell nanoparticles on the adsorption of subsequent polyelectrolytes’ layers, (**B**) size distribution of pegylated polymeric core-shell NPs.

Multilayer shell of polyelectrolyte PLL, PGA, and PGA-g-PEG was formed on PCL nanoparticles in order to prepare polymeric core-shell nanoparticles optimized for passive targeting. Since the zeta potential of PCL NPs was positive (+68 ± 3 mV), the formation of a multilayered shell was started with the polyanion PGA using the saturation technique of LbL method. Next, layers were built with PLL or PGA, and the external layer was built with pegylated-PGA. Details concerning determination of the saturation concentration for PLL, PGA and PGA-g-PEG are described in Supporting Materials (Figures S4 and S5 respectively). Figure 5A illustrates a dependence of the zeta potential of polymeric core-shell NPs on the adsorption of subsequent layers. The typical zig-zag shape can be considered as the evidence of the formation of multilayer shell. The absolute values of zeta potentials of PLL and PGA-ended polymeric core-shell nanoparticles were higher than 30 mV what provided sufficient electrostatic stabilization against aggregation or agglomeration during the layer-by-layer process. The external layer was formed with pegylated polyelectrolyte PGA-g-PEG (PGA with grafted PEG chains) and grafting of PEG on PGA decreases the charge density of polyanion, therefore, covering polymeric core-shell NPs with pegylated-PGA should significantly decrease their zeta potential in comparison with regular polyanionic layers. The measured zeta potential of pegylated polymeric core-shell NPs was close to zero (−3 ± 4 mV). Stability tests were performed to confirm that PEG corona at the polymeric nanoparticles’ surface provides sufficient steric stabilization. The average size of obtained pegylated polymeric core-shell NPs was 155 nm (PDI < 0.2) (Figure 5B), moreover, any significant changes were not observed in the size distribution and zeta potential at least 30 days. That results indicate that stable monodisperse polymeric core-shell nanoparticles can be produced under optimized condition.

The properties of our polymeric core-shell NPs make them promising candidates as a vehicle for passive targeting which based on the spontaneous drug accumulation in ‘leaky’ areas of blood vessels. In those areas with increased vascular permeability, our polymeric core-shell nanoparticles can extravasate and accumulate inside the interstitial space. It was also revealed that the surface of our polymeric core-shell NPs was functionalized by pegylation which might have the capability of avoiding the binding with immune cells or proteins in blood circulation and circulating for a long time to provide a sufficient level of target accumulation.

#### 3.3.2. Polymeric Core-Shell Nanoparticles for Magnetic Targeting

**Figure 6:**
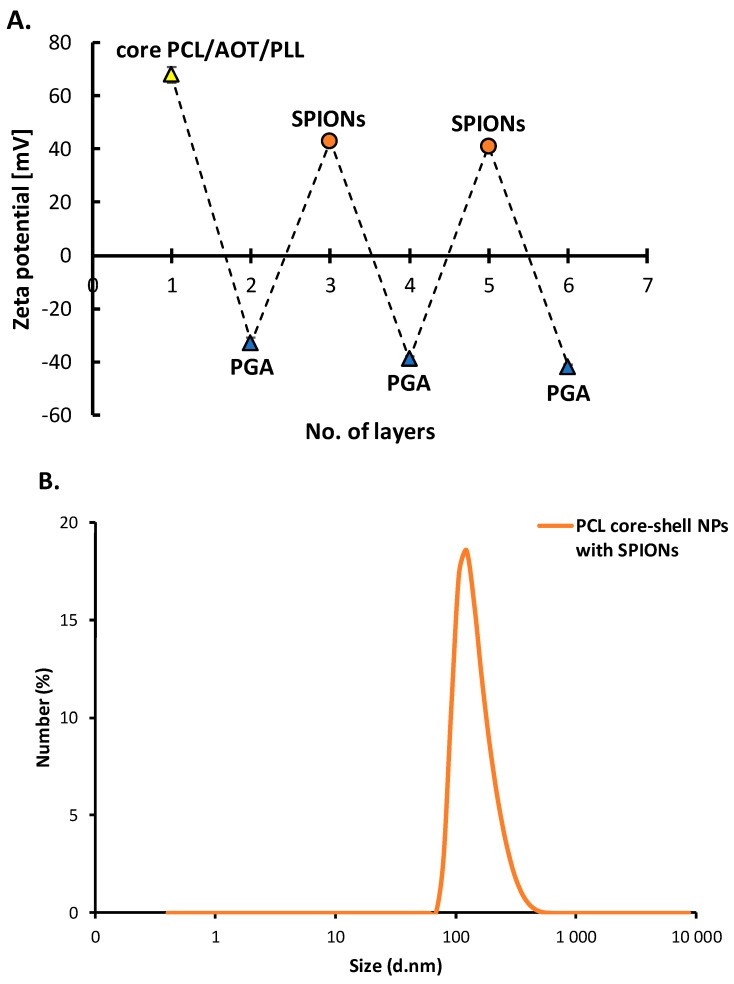
(**A**) Dependence of the zeta potential of polymeric core-shell nanoparticles on the adsorption of subsequent polyelectrolytes’ layers with superparamagnetic iron oxide nanoparticles (SPIONs), (**B**) size distribution of magnetically-responsive polymeric core-shell NPs.

A multilayer shell of polyelectrolytes PLL and PGA with embedded SPIONs was formed on PCL nanoparticles in order to prepare a magnetically-responsive nanosystem. Before the experiment, SPIONs were characterized by measurements their size (6 ± 2 nm) and zeta potential (~+50 mV). Since the zeta potential of PCL NPs was positive (+68 ± 3 mV), formation of the hybrid multilayered shell was started with polyanion PGA using the saturation technique of the LbL method. Next, layers were built with SPIONs and PGA. Details concerning determination of the saturation concentration for PGA and SPIONs are described in the Supporting Materials (Figures S4A and S6 respectively). Figure 6A illustrates a typical zig-zag dependence of the zeta potential of polymeric core-shell NPs on the adsorption of subsequent layers, which can be considered as evidence of successful incorporation of SPIONs into the multilayer shell. The absolute values of zeta potentials of formed polymeric core-shell NPs were higher than 30 mV which provided sufficient electrostatic stabilization against aggregation or agglomeration during the layer-by-layer process. The average size of the hybrid polymeric core-shell nanoparticles obtained was 160 nm (PDI < 0.3) (Figure 6B). They were stable in stock solution for a period of several weeks.

**Figure 7:**
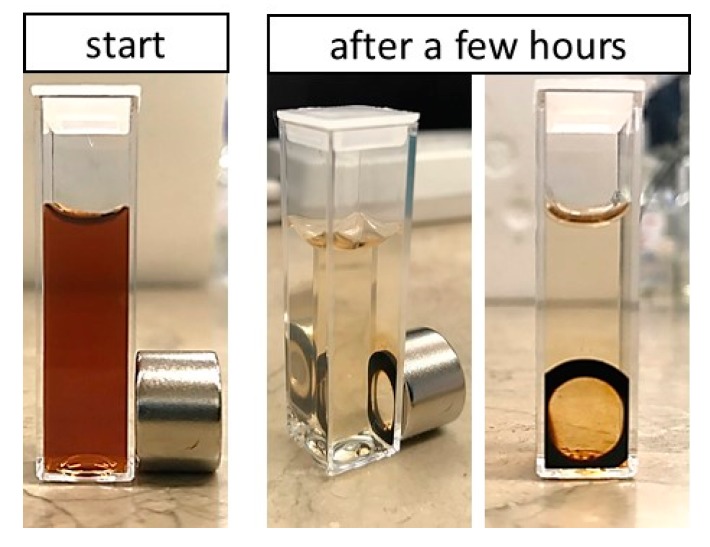
Effects of magnetic field on synthesized hybrid polymeric core-shell nanoparticles using a permanent magnet.

The proof of concept of the magnetic delivery capacity of hybrid nanoparticles is shown in Figure 7 where our hybrid polymeric core-shell NPs were attracted by the magnetic field gradient generated by a permanent magnet. The cuvette containing our magnetically-responsive polymeric core-shell nanoparticles’ suspension was placed close to a permanent magnet. In the beginning, the distribution of magnetically-responsive polymeric core-shell nanoparticles was homogeneous. After a few hours, as the result of attraction by the magnetic field, magnetically-responsive polymeric core-shell NPs were concentrated at the side of the cuvette located next to the magnet and the residual liquid became transparent. These results prove that changes in hybrid polymeric core-shell NPs’ distribution in the cuvette were caused by the magnetic field. Our magnetically-responsive hybrid polymeric core-shell nanoparticles may be very promising for future applications in magnetic drug delivery. Moreover, incorporation of SPIONs in polymeric core-shell nanoparticles opens a new perspective on the application of that systems in theranostic (MRI or hyperthermia.

#### 3.3.3. Polymeric Core-Shell Nanoparticles for Bioimaging

One of modalities that have been used for non-invasive imaging in medicine is optical imaging. From the optical imaging techniques available, those based on the fluorescence has emerged as the most powerful imaging techniques. Optical fluorescence depends on the inherent property of the fluorophore that was used for labeling. Organic fluorescent dyes are the most commonly used fluorophores. Dyes such as fluorescein isothiocyanate (FITC) and the carboxyfluorescein diacetatesuccinimidyl ester (CFSE), rhodamine (ROD) and cyanine class of dyes (NIR) have been used in various biological applications, e.g., fluorescently-labeled antibodies and molecules that are used to stain cells or organelles [39,40]. In the case of our polymeric core-shell nanoparticles, rhodamine dye was conjugated to polycation PLL and that type of fluorescently labeled polycation was used for formation of the multilayer shell.

**Figure 8:**
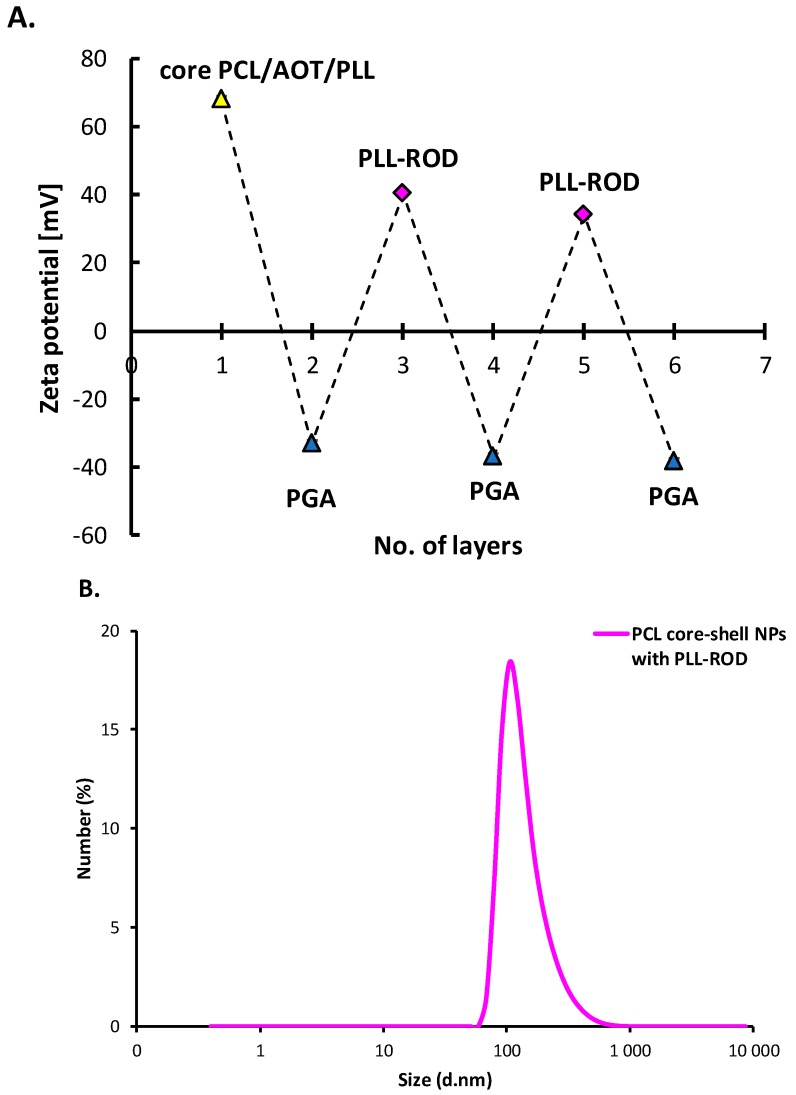
(**A**) Dependence of the zeta potential of polymeric core-shell PCL nanoparticles on the adsorption of subsequent polyelectrolytes’ layers with fluorescently-labeled polycation poly-L-lysine hydrobromide rhodamine (PLL-ROD), (**B**) size distribution of fluorescently-labeled polymeric core shell NPs.

**Figure 9:**
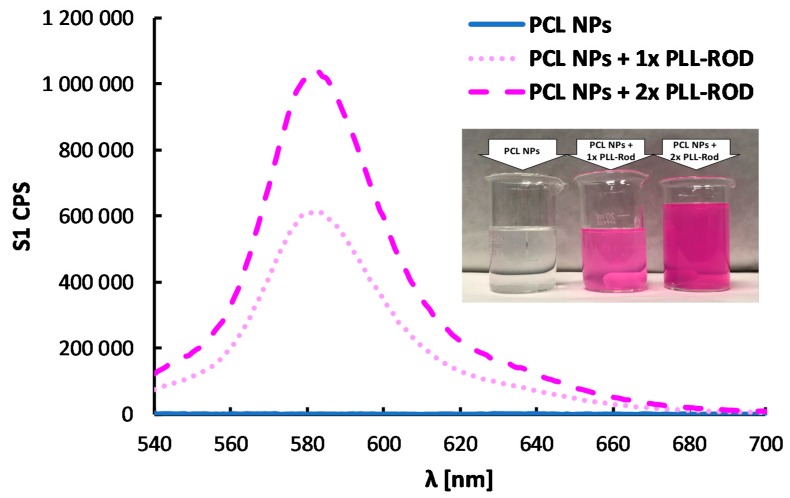
Emission spectra of fluorescently-labeled polymeric core-shell PCL nanoparticles with different number of PLL-ROD layers and a picture of the prepared suspensions.

A multilayer shell of polyanion PGA and fluorescently-labeled polycation PLL-ROD was formed on PCL nanoparticles in order to prepare fluorescently-labeled polymeric core-shell nanoparticles. Since the zeta potential of PCL NPs was positive (+68 ± 3 mV), formation of the multilayered shell was started with polyanion PGA using the saturation technique of LbL method. The next layers were built with fluorescently-labeled polycation PLL-ROD or polyanion PGA. Details concerning determination of the saturation concentration for PGA and PLL-ROD are described in the Supporting Materials (Figures S4A and S7 respectively). Figure 8A illustrates a dependence of the zeta potential of polymeric core-shell NPs on the adsorption of subsequent layers and the typical zig-zag shape can be considered as the evidence of formation of multilayer shell. Also here, the absolute values of zeta potentials of PLL-ROD and PGA-ended polymeric core-shell NPs were higher than 30 mV, which provided sufficient electrostatic stabilization against aggregation or agglomeration during the layer-by-layer process. The average size of the fluorescently labeled polymeric core-shell nanoparticles obtained was 106 nm (PDI < 0.25) (Figure 8B). Moreover, any significant changes were not observed in the size distribution and zeta potential at least 30 days. Fluorescent properties of polymeric core-shell NPs were confirmed by spectrofluorimetry, characteristic emission spectra (peak at ~580 nm) of Rhodamine can be observed and comparison of spectra of empty and loaded polymeric core-shell NPs provided evidence of the labeling of our polymeric nanoparticles (Figure 9). The results indicate that stable monodisperse fluorescently-labeled polymeric core-shell NPs could be produced under optimized condition. Incorporation of selected fluorophores in polymeric core-shell nanoparticles opens a new perspective on the application of systems in bioimaging or theranostics.

## 4. Conclusions

A new type of polymeric core-shell (PCL, PLA, PLGA) nanoparticles was developed. The nanoparticles were formed via the spontaneous emulsification solvent evaporation (SESE) method. The model hydrophobic compound (Coumarin-6) was encapsulated in our polymeric nanoparticles and, moreover, the formed polymeric NPs were further modified/functionalized/optimized for passive and magnetic targeting, as well as for bioimaging, by the layer-by-layer (LbL) method. Polymeric core-shell nanoparticles with average size ranging from 100–200 nm and low the polydispersity index (PDI) were obtained. The developed polymeric core-shell NPs may be considered as a promising platform for future nanomedicine especially in targeted drug delivery, theranostics, or bioimaging. Further work will focus on bioanalysis including biocompatibility, controlled release, and activity of the encapsulated drug, cellular uptake, localization, etc.

## Figures and Tables

**Table 1:** Polymeric nanoparticles’ characterization.

Polymer (Optimized Concentration)	Average Size	Polydispersity Index (PDI)	Zeta Potential	Concentration
Poly(caprolactone), PCL (10 mg/mL)	76 (±5) nm	0.134 (±0.027)	68 (±3) mV	~1 × 10^11^ nanoparticle/mL
Poly(lactic acid), PLA (2.5 mg/mL)	80 (±7) nm	0.166 (±0.021)	71 (±4) mV	~1 × 10^11^ nanoparticle/mL
Poly(lactide-co-glycolide), PLGA (5 mg/mL)	77 (±2) nm	0.179 (±0.048)	78 (±2) mV	~1 × 10^11^ nanoparticle/mL

**Table 2:** Encapsulation efficacy of Coumarin-6 loaded polymeric nanoparticles (NPs).

	Optimized Coumarin-6 Concentration in the Oil Phase	Final Coumrine-6 Concentration in Nanoparticles’ Suspension	Encapsulation Efficiency	Polymer/drug Ratio
PCL	0.3 mg/mL	0.150 mg/L	98.9%	33
PLA	0.25 mg/mL	0.125 mg/L	98.7%	10
PLGA	0.25 mg/mL	0.125 mg/L	98.8%	20

## Supplementary Materials

The following are available online at https://www.mdpi.com/2079-4991/10/3/496/s1, Figure S1: Chemical structures of polymers and dyes. Figure S2: Changes of the zeta potential of emulsion cores with the AOT/PLL ratio. Figure S3: Emission spectra of Coumarin-6 loaded polymeric nanoparticles and their filtrates after ultracentrifugation. Figure S4: Changes of the zeta potential during formation of the (A) PGA layer (B) PLL layer. The condition marked by an asterisk on both figures represents the first stable sample after overcharging. Figure S5: Changes of the zeta potential during formation of the PGA-g-PEG layer. The condition marked by an asterisk represents the first stable sample after overcharging. Figure S6: Changes of the zeta potential during formation of the SPIONs layer. The condition marked by an asterisk represents the first stable sample after overcharging. Figure S7: Changes of the zeta potential during formation of the PLL-ROD layer. The condition marked by an asterisk represents the first stable sample after overcharging. Table S1: Optimized parameters for nanoparticle’ synthesis.
