# Supplementary material for: Polymeric Core-Shell Nanoparticles Prepared by Spontaneous Emulsification Solvent Evaporation and Functionalized by the Layer-by-Layer Method
Source: Nanomaterials (Basel). 2020 Mar 10;10(3):496. doi: 10.3390/nano10030496 (PMC7153481; doi:10.3390/nano10030496)
Supplement: Supplementary file 1 [file nanomaterials-10-00496-s001.pdf]

Supplementary Information

Polymeric Core-Shell Nanoparticles Prepared by Spontaneous Emulsification Solvent Evaporation and Functionalized by the Layer-by-Layer Method

Marta Szczęch, Krzysztof Szczepanowicz*

Jerzy Haber Institute of Catalysis and Surface Chemistry, Polish Academy of Sciences, Krakow, Poland

* Correspondence: Krzysztof Szczepanowicz

Tel.: +48126395121

Fax: +48124251923

E-mail: ncszczep@cyf-kr.edu.pl

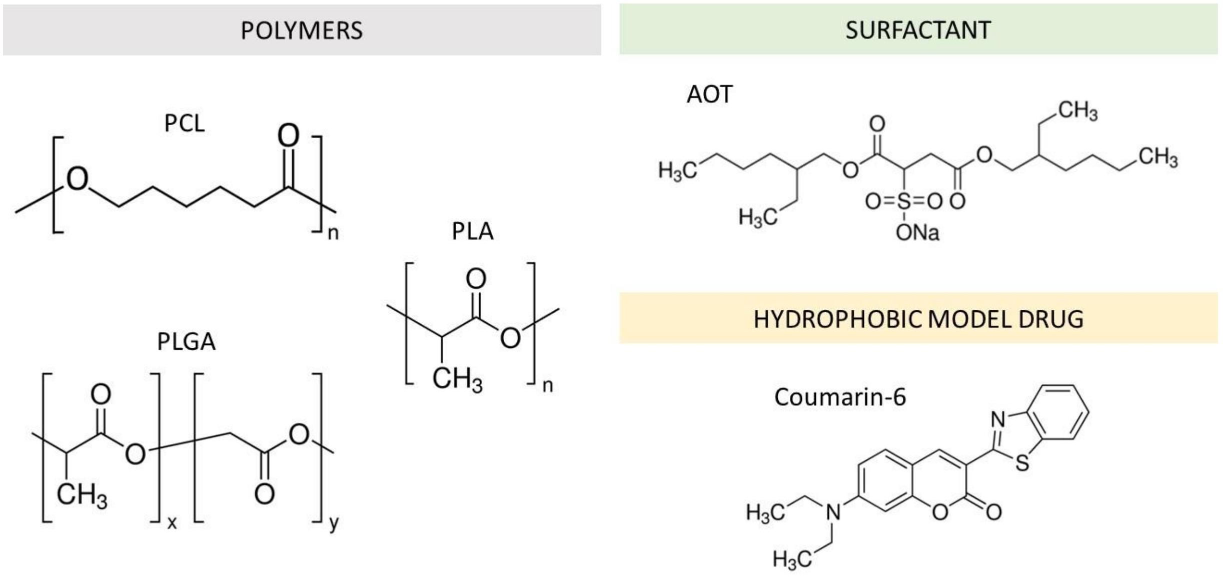

Figure S1. Chemical structures of polymers and dyes.

Table S1. Optimized parameters for nanoparticles' synthesis.

Drug-loaded NPs' synthesis	Oil phase (0.1 ml)	Water phase (200 ml)
**PCL/C-6 NPs**	PCL 1 mg, AOT 33 mg, Coumarin-6 0.03 mg	PLL (200 ppm)
**PLA/C-6 NPs**	PLA 0.25 mg, AOT 33 mg, Coumarin-6 0.025 mg	PLL (200 ppm)
**PLGA/C-6 NPs**	PLGA 0.5 mg, AOT 33 mg, Coumarin-6 0.025 mg	PLL (200 ppm)

Optimization of the nanoemulsion's formation stabilized by AOT/PLL interfacial complex

For determination of the optimal oil phase/polycation ratio, the concentration of PLL was varied from 10 to 250 ppm, while a volume of the oil phase was fixed. The optimal amount of PLL was determined by measuring the zeta potential of formed nanodroplets and it corresponds to the point just before reaching the plateau of dependence of the zeta potential on the added amount of PLL. The optimal volume is marked by an asterisk (Figure S2) indicating conditions when the unadsorbed PLL in the polymeric nanoparticles' suspension was minimized, as most of it was used to form the AOT/PLL interfacial complex.

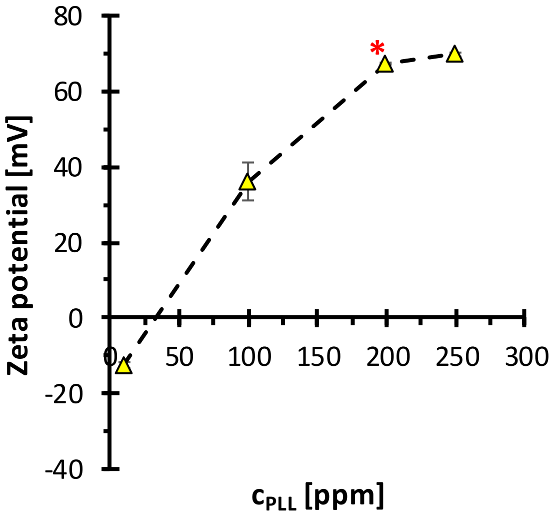

Figure S2. Changes of the zeta potential of emulsion cores with the AOT/PLL ratio.

The evidence of the model substance (Coumarin-6) encapsulation into polymeric NPs

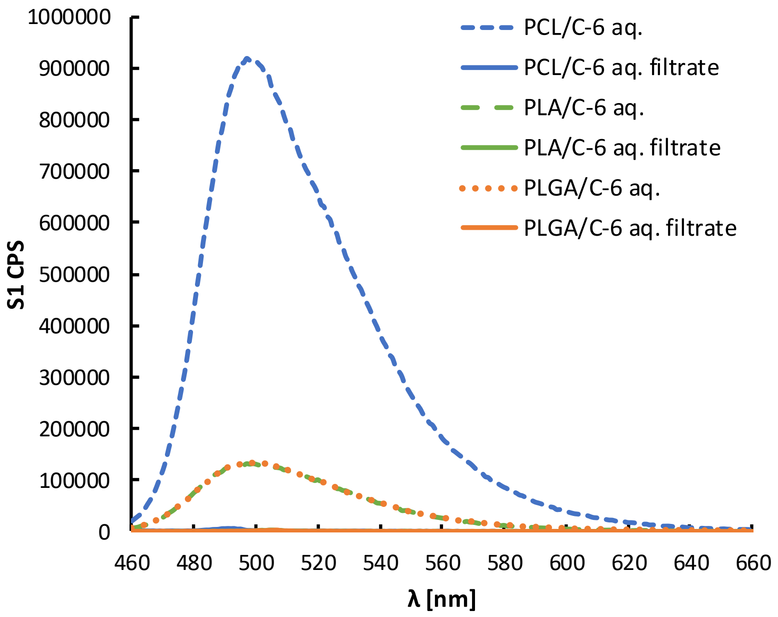

Figure S3. Emission spectra of Coumarin-6 loaded polymeric nanoparticles and their filtrates after ultracentrifugation.

Determination of saturation concentration of PLL, PGA and PGA-g-PEG

PGA and PLL

Fixed volume (10 ml) of polymeric nanoparticles' suspension was added under mixing to the oppositely charged polyelectrolyte solution (PGA). Volumes of the polyelectrolyte solution used to form the saturated layer were chosen empirically by measuring the zeta potential of formed polymeric core-shell nanoparticles. The optimal volume corresponds to the point just before reaching the plateau of dependence of the zeta potential on the added amount of PGA. This volume is marked by an asterisk (Figure S4A) indicating conditions when the zeta potential of formed polymeric core-shell nanoparticles reached a value, close to the zeta potential of the PGA in solution. By this approach, the unadsorbed PGA in the polymeric core-shell nanoparticles' suspension was minimized, as most of it was used to form polyelectrolyte layer on polymeric core-shell nanoparticles, which resulted in the nanoparticles' surface charge reversal. Then, the next layer in the shell's structure was built from polycation PLL. Fixed volume of PGA-terminated polymeric core-shell nanoparticles was added to the PLL solution under mixing. Volume of the PLL solution used to form the saturated layer was chosen empirically and it corresponds to the point just before reaching the plateau of dependence of

the zeta potential on the added volume of PLL solution. The optimal volume is again marked by an asterisk (Figure S4B) and it indicates the conditions when the zeta potential of formed polymeric core-shell nanoparticles reached a value, close to the zeta potential of the PLL in solution. The procedure described above was repeated until desired number of layers was formed. Optimized volumes of PGA and PLL used for the formation of consecutive layers of the shell were as follows: 1 ml PGA, 1.2 ml PLL, 1.5 ml PGA, 3.3 ml PLL, 4.0 ml PGA (polyelectrolytes’ concentration 2 mg/ml).

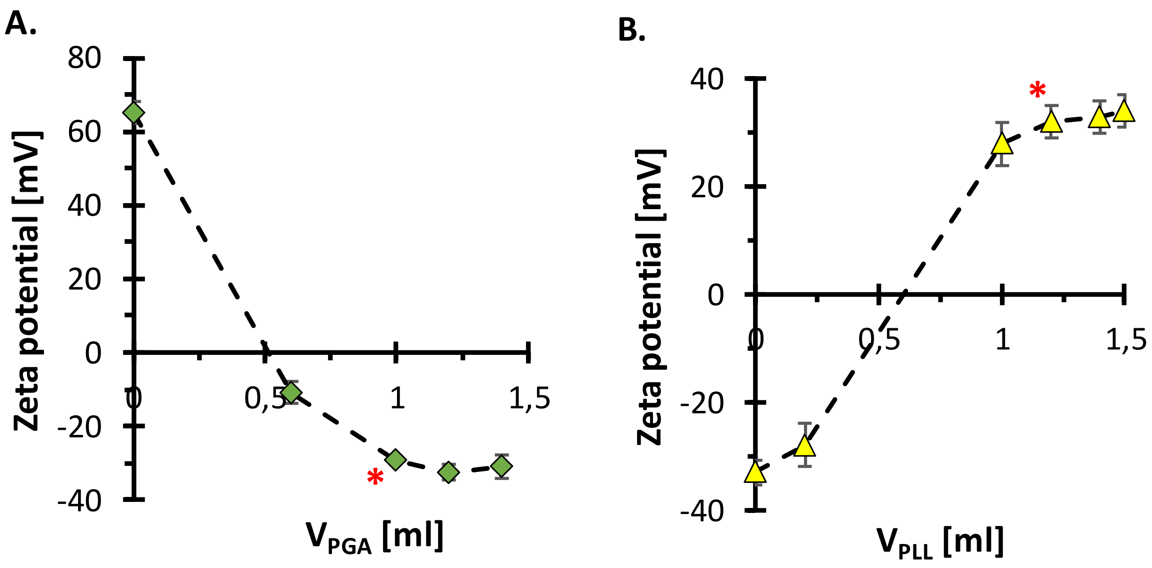

Figure S4. Changes of the zeta potential during formation of the (A) PGA layer (B) PLL layer. The condition marked by an asterisk on both figures represents the first stable sample after overcharging.

PGA-g-PEG

For the preparation of pegylated polymeric core-shell nanoparticles, positively charged PLL-terminated polymeric core-shell nanoparticles were coated with previously synthesized pegylated polyanion PGA-g-PEG [1]. The saturation concentration of PGA-g-PEG was determined using the same procedure as for a regular polyelectrolyte. The optimal volume of PGA-g-PEG used to form the stable pegylated external layer corresponds to the point just before reaching the plateau of dependence of the zeta potential on the added amount of PGA-g-PEG and is marked by an asterisk in Figure S5. The optimized volume of PGA-g-PEG was 17.5 ml (PGA-g-PEG concentration 200 mg/ml).

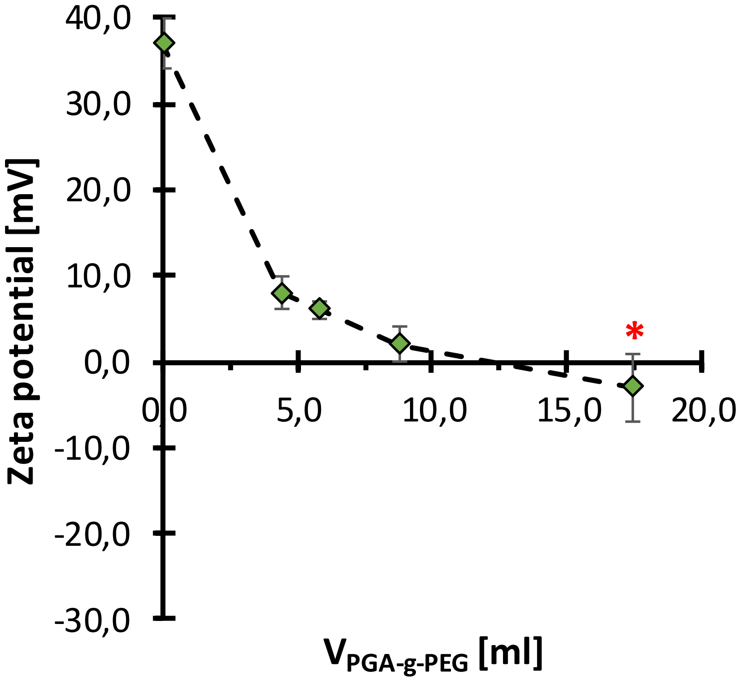

Figure S5. Changes of the zeta potential during formation of the PGA-g-PEG layer. The condition marked by an asterisk represents the first stable sample after overcharging.

Determination of saturation concentration for PGA and SPIONs

The PGA layer was built as described above, while the next layer in the shell structure was built from SPIONs (Superparamagnetic Iron Oxide Nanoparticles) using a procedure similar to that described for the polyelectrolyte. The fixed volume of PGA-terminated polymeric core-shell nanoparticles was added to SPIONs' suspension during continuous shaking. The volume of SPIONs' suspension used to form the saturated layer was chosen empirically and it corresponds to the point just before reaching the plateau of dependence of the zeta potential on the added volume of SPIONs. The optimal volume of SPIONs is again marked by an asterisk on Figure S6 indicating conditions when the zeta potential of formed polymeric core-shell nanoparticles reached the value close to the zeta potential of SPIONs nanoparticles in solution. By this method, the amount of non-adsorbed SPIONs in the polymeric core-shell nanoparticles' suspension was minimized. Next hybrid layers were formed by repeating the procedure described above. The optimized volumes of PGA and SPIONs used for the formation of consecutive layers of the shell were as follows: 1.5 ml PGA, 3.45 ml SPIONs, 17.25 ml PGA, 3.5 ml SPIONs, 40.25 ml PGA (PGA concentration 2 mg/ml, while SPIONs (aq.) 5% wt.).

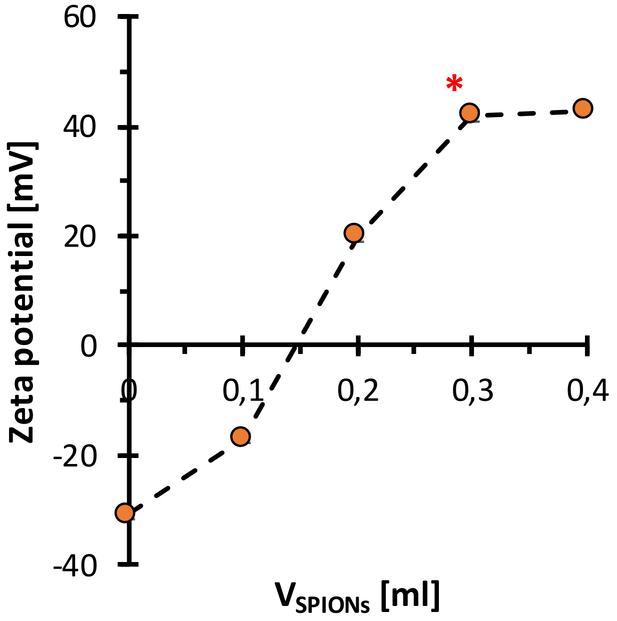

Figure S6. Changes of the zeta potential during formation of the SPIONs layer. The condition marked by an asterisk represents the first stable sample after overcharging.

Determination of saturation concentration for PGA and PLL-ROD

The PGA layer was built as described above. Then, the next layer in the shell structure was built from fluorescently-labeled polycation PLL-ROD. Fixed volume of PGA-terminated polymeric core-shell nanoparticles was added to PLL-ROD solution under mixing. The volume of PLL-ROD solution used to form the saturated layer was chosen empirically and it corresponds to the point just before reaching the plateau of dependence of the zeta potential on the added volume of PLL-ROD solution. The optimal volume is again marked by an asterisk (Figure S7) indicating conditions when the zeta potential of formed polymeric core-shell nanoparticles reached the value close to the zeta potential of the PLL-ROD in solution. This procedure was repeated until the desired number of layers was formed. The optimized volumes of PGA and PLL-ROD used for the formation of consecutive layers of the shell were as follows: 1 ml PGA, 2 ml PLL-ROD, 1.5 ml PGA, 3 ml PLL-ROD, 4.5 ml PGA (all polyelectrolytes' concentration 2 mg/ml).

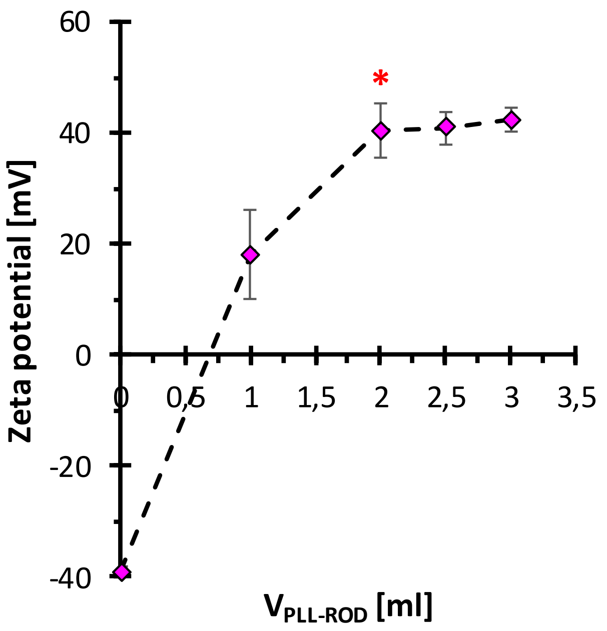

Figure S7. Changes of the zeta potential during formation of the PLL-ROD layer. The condition marked by an asterisk represents the first stable sample after overcharging.

References:

1. S. Lukasiewicz, K. Szczepanowicz, E. Blasiak, M. Dziedzicka-Wasylewska, *Langmuir* **2015**, *31*, 6415-6425.
